# Supplementary material for: Development of Polymer Composites Using Surface-Modified Olive Pit Powder for Fused Granular Fabrication
Source: Polymers (Basel). 2024 Oct 24;16(21):2981. doi: 10.3390/polym16212981 (PMC11548441; doi:10.3390/polym16212981)
Supplement: Supplementary file 1 [file polymers-16-02981-s001.zip › polymers-3256334-supplementary.pdf]

Supplementary Materials

Development of Polymer Composites Using Surface-Modified Olive Pit Powder for Fused Granular Fabrication

Table S1. Different printing temperatures tested for PETG5OPP by FGF. The third value listed in the "FGF extruder temperature profile" column is used in the main text as the printing temperature.

FGF Extruder Temperature Profile (°C)	Remarks
230/235/240	Material degradation, irregular and foamy finish.
220/225/230	Material degradation, irregular and foamy finish.
210/215/220	Material degradation.
200/205/210	Printed without problems, uniform printed bead.
195/200/205	Clogging.

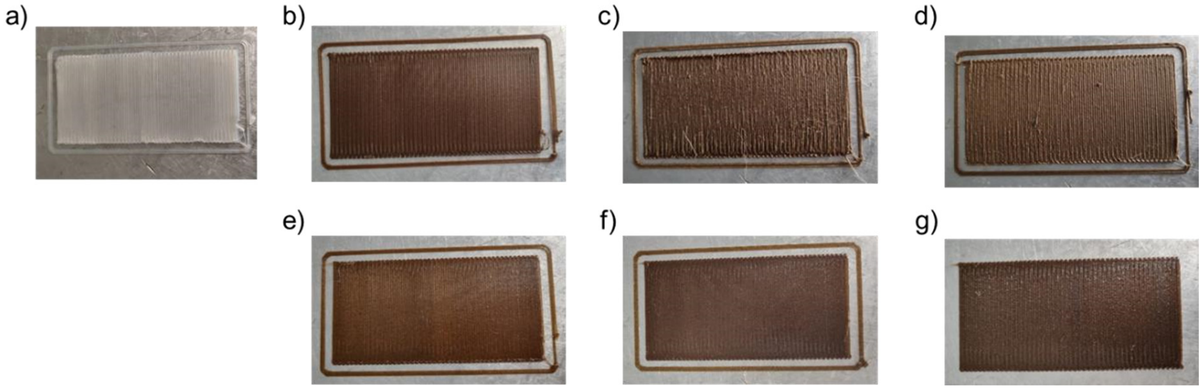

Figure S1. Digital images of (**a**) PETG; (**b**) PETG5OPP; (**c**) PETG10OPP; (**d**) PETG15OPP; (**e**) PETG5MOPP; (**f**) PETG10MOPP and (**g**) PETG15MOPP XY plates.

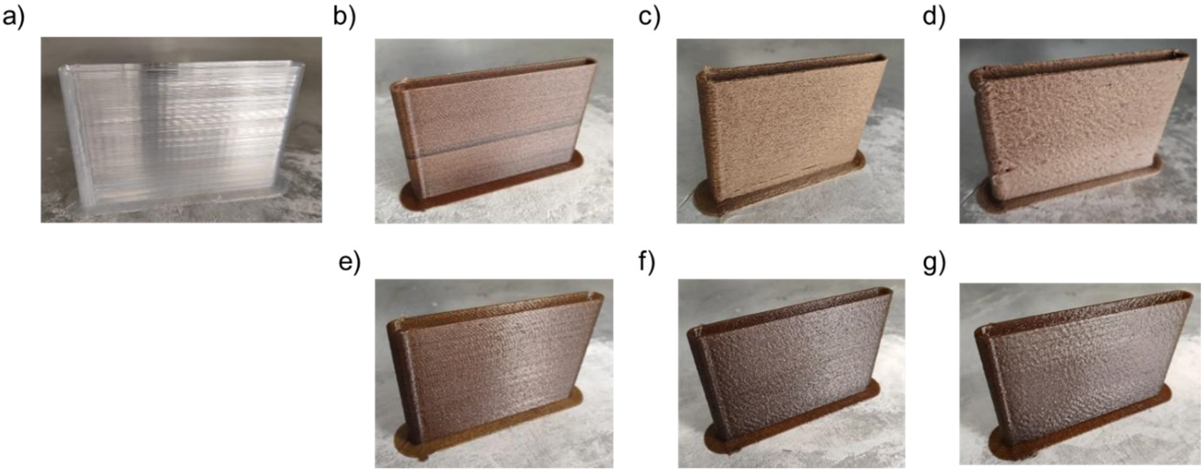

Figure S2. Digital images of (**a**) PETG; (**b**) PETG5OPP; (**c**) PETG10OPP; (**d**) PETG15OPP; (**e**) PETG5MOPP; (**f**) PETG10MOPP and (**g**) PETG15MOPP XZ plates.

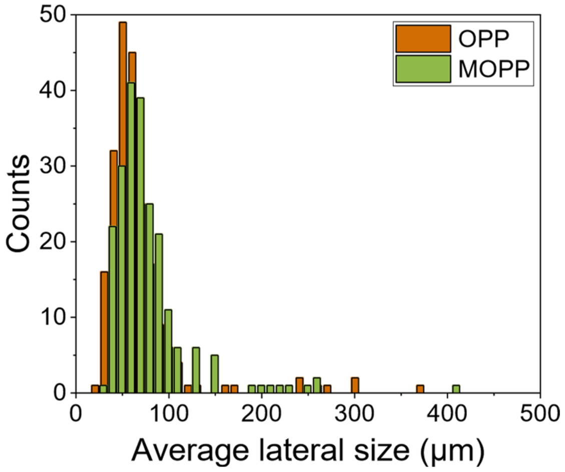

Figure S3. Particle size distribution of OPP and MOPP.

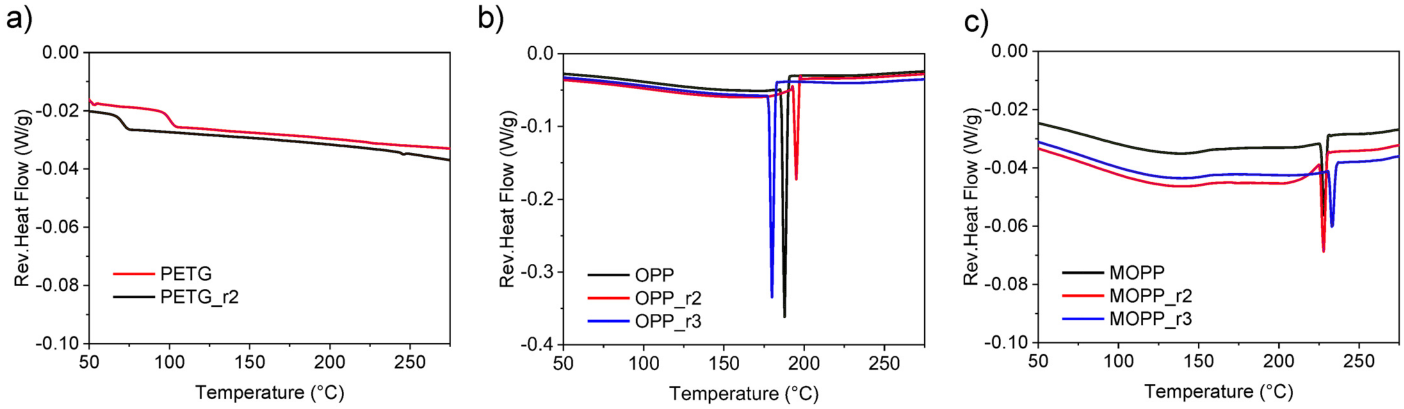

Figure S4. Reversing heat flow curves of (**a**) PETG; (**b**) OPP and (**c**) MOPP.

Table S2. Young's modulus, tensile strength and elongation at break values from tensile testing curves of XY and XZ specimens manufactured by FGF.

	Young's Modulus (MPa)		Tensile Strength (MPa)		Elongation at Break (%)	
**Material**	**FGF_XY**	**FGF_XZ**	**FGF_XY**	**FGF_XZ**	**FGF_XY**	**FGF_XZ**
PETG	1710 ± 60	1320 ± 50	45.6 ± 1.2	20.5 ± 1.4	110 ± 30	1.7 ± 0.1
PETG5OPP	1640 ± 90	1010 ± 30	35.5 ± 2.4	15.5 ± 0.6	3.4 ± 0.2	1.8 ± 0.1
PETG10OPP	1170 ± 60	450 ± 40	19.0 ± 1.3	6.0 ± 0.3	2.7 ± 0.2	2.5 ± 0.6
PETG15OPP	920 ± 50	240 ± 30	13.1 ± 0.8	3.4 ± 0.3	2.7 ± 0.3	2.0 ± 0.4
PETG5MOPP	1890 ± 60	1420 ± 50	41.2 ± 1.0	10.9 ± 1.0	3.2 ± 0.1	0.8 ± 0.1
PETG10MOPP	2040 ± 100	1380 ± 20	28.4 ± 2.4	7.6 ± 0.6	1.5 ± 0.1	0.5 ± 0.1
PETG15MOPP	1800 ± 60	1650 ± 140	12.2 ± 2.2	8.7 ± 2.3	0.7 ± 0.1	0.6 ± 0.2

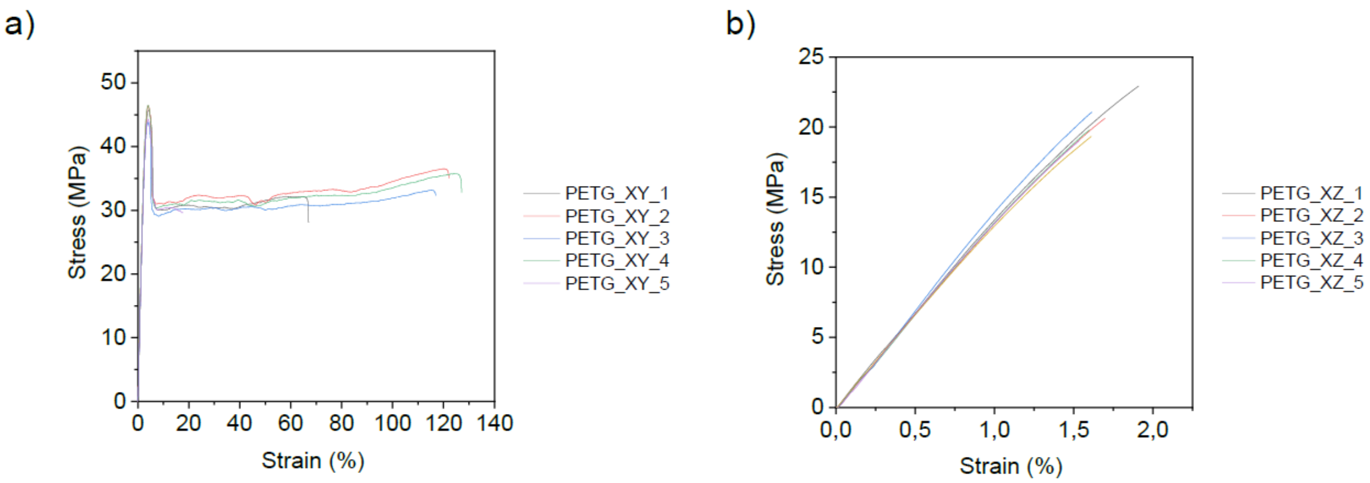

Figure S5. Tensile testing curves of all the individual repeats tested for (**a**) PETG FGF XY and (**b**) PETG FGF XZ.

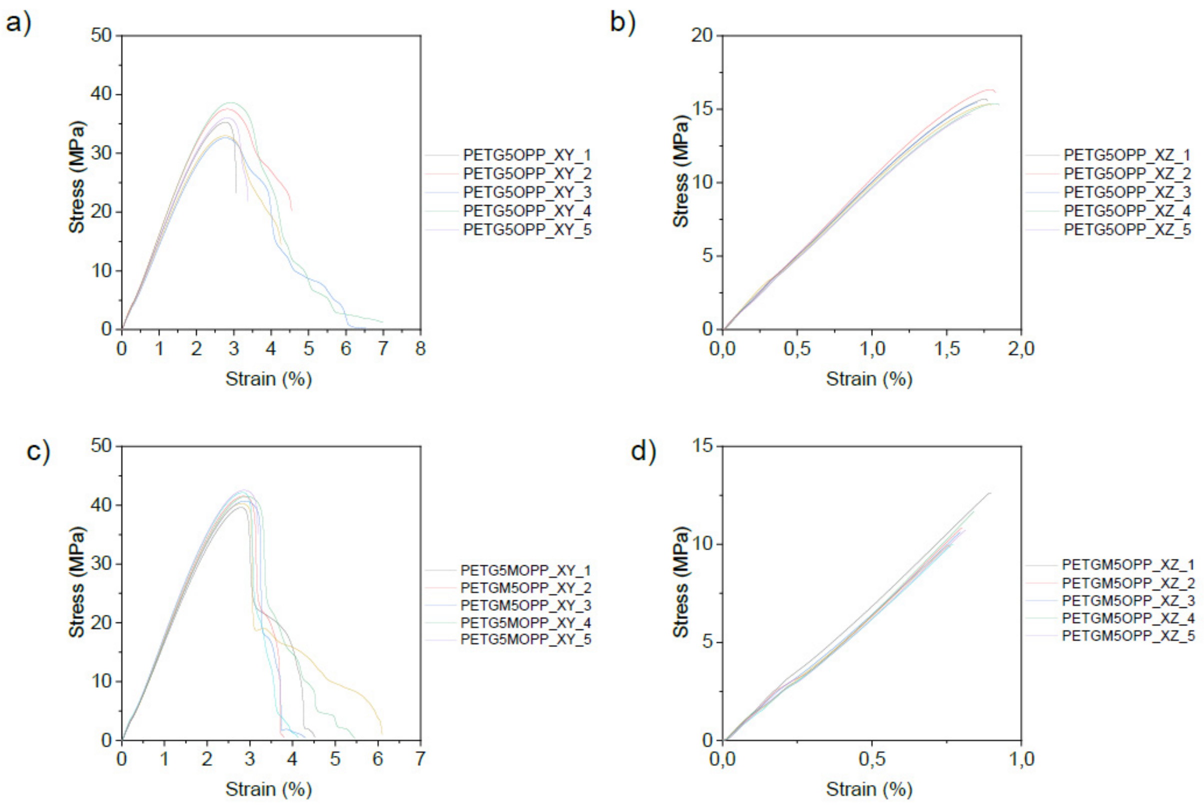

Figure S6. Tensile tesing curves of all the individual repeats tested for (**a**) PETG5OPP FGF XY (**b**) PETG5OPP FGF XZ (**c**) PETG5MOPP FGF XY (**d**) PETG5MOPP FGF XZ samples.

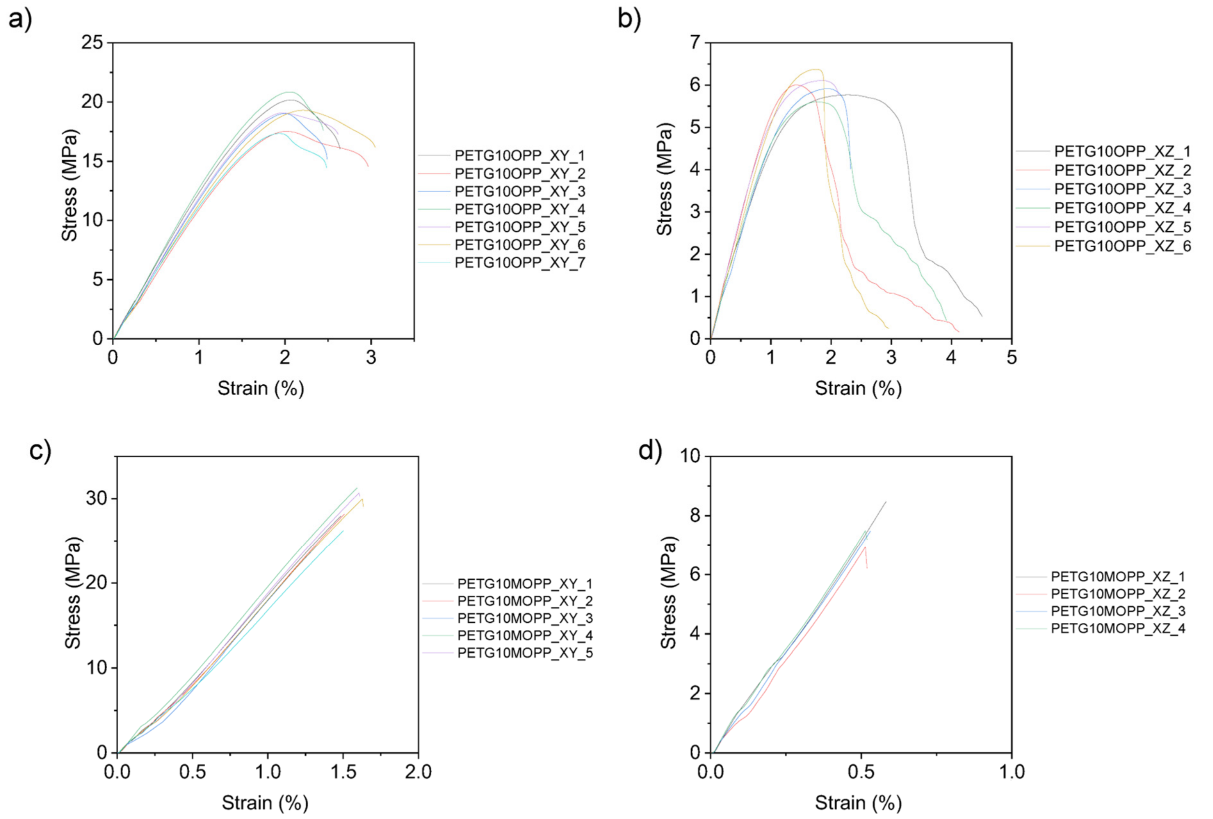

Figure S7. Tensile testing curves of all the individual repeats tested for (**a**) PETG10OPP FGF XY (**b**) PETG10OPP FGF XZ (**c**) PETG10MOPP FGF XY and (**d**) PETG10MOPP FGF XZ samples.

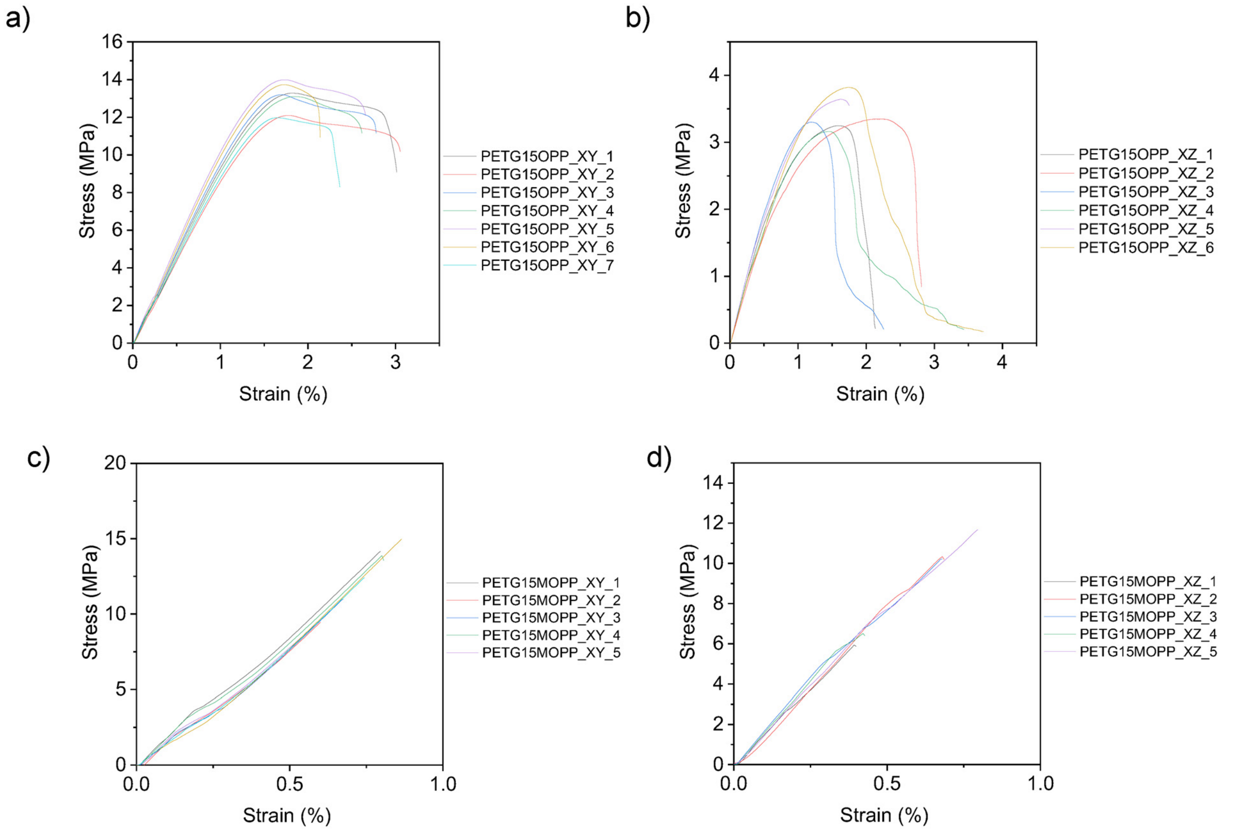

Figure S8. Tensile testing curves of all the individual repeats tested for (**a**) PETG15OPP FGF XY (**b**) PETG15OPP FGF XZ (**c**) PETG15MOPP FGF XY and (**d**) PETG15MOPP FGF XZ samples.

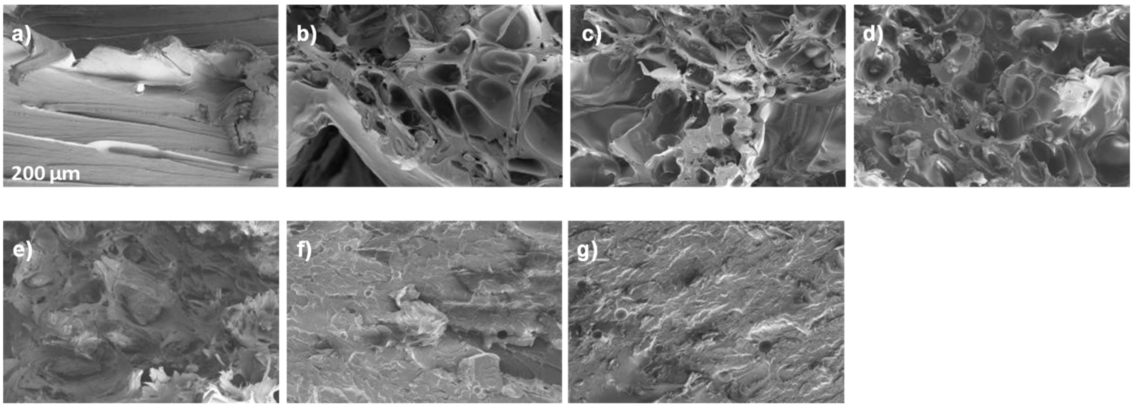

Figure S9. SEM images of the fracture surface of XY tensile testing specimens of (**a**) PETG; (**b**) PETG5OPP; (**c**) PETG10OPP; (**d**) PETG15OPP; (**e**) PETG5MOPP; (**f**) PETG10MOPP and (**g**) PETG15MOPP. Scale bar in (**a**) is applicable to all images.
